# Rickettsioses and Q Fever in Tanzania: Estimating the Burden of Pervasive and Neglected Causes of Severe Febrile Illness in Sub-Saharan Africa

**DOI:** 10.4269/ajtmh.21-0963

**Published:** 2021-12-20

**Authors:** Paul W. Blair, Mohammed Lamorde, J. Stephen Dumler

**Affiliations:** ^1^ Department of Pathology, Uniformed Services University, Bethesda, Maryland;; ^2^ Infectious Diseases Institute, Makerere University, Kampala, Uganda

Rickettsial infections are well-described as second only to malaria as sources of fever among travelers returning from sub-Saharan Africa (SSA).^1^ Studies of the epidemiology of spotted fever group *Rickettsia* (SFGR) and *Coxiella burnetii* (Q fever) infections have otherwise been limited, suggesting that these travel-related infections could be sentinel events to a largely undetected burden of rickettsial infections in SSA.^1^^–^^3^ Although the distribution of SFGR species causing infections in SSA, including *Rickettsia africae* and *Rickettsia conorii*, is largely unknown, the geographic distribution of *Amblyomma* tick vectors spans most of the continent, and infections caused by *R. africae* transmitted by *Amblyomma* tick bites could be common throughout SSA.^4^ Additionally, although the morbidity and mortality caused by SFGR in SSA are largely unknown, a fatal case was discovered in Kenya,^5^ and *Rickettsia spp.* were identified as the cause of sepsis in Uganda.^6^ Therefore, rickettsial infections are clearly a problem in SSA; however, the degree of the burden represents a nonmalarial febrile illness knowledge gap.

In this issue of the *American Journal of Tropical Medicine and Hygiene*, Pisharody, et al. estimate the incidences of SFGR and Q fever in Tanzania.^7^ Rickettsial infection incidence estimates were previously limited by the need for population-wide demographic information to adjust estimates for time and population. Pisharody et al. built on prior cohort research to estimate the incidences of SFGR and Q fever by surveying households in the region served by a healthcare facility to understand healthcare utilization and community demographics.^7^ This is the first study to estimate the overall incidence of infections caused by *Rickettsia* spp. and *C. burnetii* in SSA. These infections were previously estimated to cause between 2% and 9% of undifferentiated febrile illnesses among hospitalized people in SSA.^8^^,^^9^ Consistent with the estimates determined in Kenya as 22% of outpatient febrile children with SFGR and 9% with Q fever,^10^ Pisharody et al. also identified a markedly higher incidence among children than adults. Although this article is a useful step toward understanding the epidemiology of SFGR and Q fever, accurate and low-cost diagnostics are needed to facilitate surveillance systems to understand broader trends and regional differences in the incidence of zoonotic and arthropod-borne bacterial infections, including SFGR and Q fever in SSA.

Our understanding of the epidemiology of SFGR and Q fever is severely restricted by the lack of clinician recognition of these illnesses and the paucity of point-of-care diagnostics in both low-resource and high-resource settings. Bacterial zoonoses including Q fever and SFGR are not commonly considered by clinicians.^11^ Eschars as early clues to SFGR diagnosis may be ignored, absent, or hidden under clothing.^12^ Although SFGR and Q fever are uniformly treatable with doxycycline, empiric regimens for febrile illness generally do not include doxycycline, which is a low-cost and widely available antibiotic in SSA. Therefore, education, surveillance, and clinical research are needed to increase awareness among clinicians and public health leaders. Clinical trials involving doxycycline in SSA could determine the optimal use of the drug and lead to improved outcomes for nonmalarial febrile illnesses.

The limited clinical recognition of SFGR or Q fever is exacerbated by the absence of rapid and accurate diagnostic tests with the ability to discriminate between closely related pathogens. The most available rapid test, the Weil–Felix agglutination test, is severely limited by poor performance characteristics for SFGR.^13^ Diagnostic testing using the gold standard of paired acute and convalescent immunofluorescence assay (IFA) serology conducted by the CDC Division of Vector-Borne Diseases, Rickettsial Zoonoses Branch (mentioned in Pisharody, et al.) is not generally available at hospitals in East Africa, and polymerase chain reaction (PCR) approaches are limited to research laboratories. When testing is available, the utility of IFA is limited in an acute care setting. A positive serologic test lags behind the onset of symptoms by at least 1 week. Single acute-phase samples for IgM lack specificity. To obtain improved specificity, a four-fold increase in titers at 1 month follow-up is required, but this cannot guide acute treatment. Moreover, IFA is incapable of clearly delineating the causative SFGR agent, thus complicating the interpretation of both clinical and surveillance studies. Pisharody et al. tested part of their cohort for antibodies to *R. conorii* and part of their cohort for *R. africae*, two distinct SFGR agents that cause diseases of very different severity yet provide indistinguishable IFA results. The impracticality and limited availability of modern diagnostic tools preserve and exacerbate the poor clinical and public health recognition of rickettsial infections in SSA.

Alternative diagnostic strategies are available at a few reference laboratories, including PCR of whole blood, eschar biopsies, or eschar swabs, which are highly specific.^14^ Multiplex PCR^15^ or TaqMan Array Cards are used in cohort studies to test multiple targets,^6^ but these are not validated for clinical use, and sensitivity is suboptimal with whole blood samples, thus limiting clinical utility.^16^ The low clinical sensitivity of PCR compared with paired serology also leads to underestimates of febrile illness caused by rickettsiae and explains lower detection rates among prior analogous populations.^17^ For example, PCR in a pediatric acute febrile illness study in Zanzibar, Tanzania, did not detect any evidence of rickettsial infection in 83 children tested for SFGR by PCR. The established poor sensitivity of SFGR PCR makes conclusions about SFGR burden challenging. Approaches to optimize sensitivity include using buffy coat or direct eschar testing.^15^ The development and production of more sensitive and validated molecular assays offer the potential to address both surveillance and diagnostic challenges. Until then, additional epidemiologic studies using serial IFA are required to understand the epidemiology and impact of febrile illnesses caused by SFGR and Q fever in SSA.

## References

[1] Leder K , 2013. GeoSentinel surveillance of illness in returned travelers, 2007–2011. Ann Intern Med 158: 456–468. 23552375 10.7326/0003-4819-158-6-201303190-00005 PMC4629801

[2] Jensenius M Fournier PE Vene S Hoel T Hasle G Henriksen AZ Hellum KB Raoult D Myrvang B, 2003. African tick bite fever in travelers to rural sub-Equatorial Africa. Clin Infect Dis 36: 1411–1417. 12766836 10.1086/375083

[3] Cherry CC Denison AM Kato CY Thornton K Paddock CD, 2018. Diagnosis of spotted fever group rickettsioses in U.S. travelers returning from Africa, 2007–2016. Am J Trop Med Hyg 99: 136–142. 29848404 10.4269/ajtmh.17-0882 PMC6085799

[4] Jensenius M Fournier PE Kelly P Myrvang B Raoult D, 2003. African tick bite fever. Lancet Infect Dis 3: 557–564. 12954562 10.1016/s1473-3099(03)00739-4

[5] Rutherford JS Macaluso KR Smith N Zaki SR Paddock CD Davis J Peterson N Azad AF Rosenberg R, 2004. Fatal spotted fever rickettsiosis, Kenya. Emerg Infect Dis 10: 910–913. 15200829 10.3201/eid1005.030537 PMC3323220

[6] Moore CC Jacob ST Banura P Zhang J Stroup S Boulware DR Scheld WM Houpt ER Liu J, 2019. Etiology of sepsis in Uganda using a quantitative polymerase chain reaction-based TaqMan Array Card. Clin Infect Dis 68: 266–272. 29868873 10.1093/cid/ciy472 PMC6321855

[7] Pisharody M Sr , 2022. Incidence of acute Q fever and spotted fever group rickettsioses, Kilimanjaro, Tanzania, 2007–2008 and 2012–2014. Am J Trop Med Hyg 106: 494–503. 10.4269/ajtmh.20-1036 PMC8832940 34929672

[8] Vanderburg S Rubach MP Halliday JE Cleaveland S Reddy EA Crump JA, 2014. Epidemiology of *Coxiella burnetii* infection in Africa: a OneHealth systematic review. PLoS Negl Trop Dis 8: e2787. 24722554 10.1371/journal.pntd.0002787 PMC3983093

[9] Crump JA , 2013. Etiology of severe non-malaria febrile illness in northern Tanzania: a prospective cohort study. PLoS Negl Trop Dis 7: e2324. 23875053 10.1371/journal.pntd.0002324 PMC3715424

[10] Maina AN Farris CM Odhiambo A Jiang J Laktabai J Armstrong J Holland T Richards AL O’Meara WP, 2016. Q fever, scrub typhus, and rickettsial diseases in children, Kenya, 2011–2012. Emerg Infect Dis 22: 883–886. 27088502 10.3201/eid2205.150953 PMC4861507

[11] Njeru J Henning K Pletz MW Heller R Forstner C Kariuki S Fèvre EM Neubauer H, 2016. Febrile patients admitted to remote hospitals in northeastern Kenya: seroprevalence, risk factors and a clinical prediction tool for Q-fever. BMC Infect Dis 16: 244. 27260261 10.1186/s12879-016-1569-0 PMC4891891

[12] Raoult D , 2001. *Rickettsia* africae, a tick-borne pathogen in travelers to sub-Saharan Africa. N Engl J Med 344: 1504–1510. 11357153 10.1056/NEJM200105173442003

[13] Hechemy KE Stevens RW Sasowski S Michaelson EE Casper EA Philip RN, 1979. Discrepancies in Weil-Felix and microimmunofluorescence test results for Rocky Mountain spotted fever. J Clin Microbiol 9: 292–293. 107194 10.1128/jcm.9.2.292-293.1979 PMC273012

[14] Mouffok N Socolovschi C Renvoise A Parola P Raoult D, 2011. Diagnosis of rickettsioses from eschar swab samples, Algeria. Emerg Infect Dis 17: 1968–1969. 22000389 10.3201/eid1710.110332 PMC3310672

[15] Reller ME Dumler JS, 2020. Optimization and evaluation of a multiplex quantitative PCR assay for detection of nucleic acids in human blood samples from patients with spotted fever rickettsiosis, typhus rickettsiosis, scrub typhus, monocytic ehrlichiosis, and granulocytic anaplasmosis. J Clin Microbiol 58: e01802–e01819. 32493778 10.1128/JCM.01802-19 PMC7448621

[16] Paris DH Dumler JS, 2016. State of the art of diagnosis of rickettsial diseases: the use of blood specimens for diagnosis of scrub typhus, spotted fever group rickettsiosis, and murine typhus. Curr Opin Infect Dis 29: 433–439. 27429138 10.1097/QCO.0000000000000298 PMC5029442

[17] Hercik C , 2017. A diagnostic and epidemiologic investigation of acute febrile illness (AFI) in Kilombero, Tanzania. PLoS One 12: e0189712. 29287070 10.1371/journal.pone.0189712 PMC5747442

